# Transcriptomic monitoring of Douglas-fir heartwood formation

**DOI:** 10.1186/s12863-023-01172-z

**Published:** 2023-11-20

**Authors:** Didier Delourme, Laure Brémaud, Idelette Plazanet, Patrick Pélissier, Philippe Label, Nathalie Boizot, Christian Breton, Stéphanie Durand, Guy Costa

**Affiliations:** 1 Université de Limoges, LABCIS, UR 22722, 123 avenue Albert Thomas, Limoges, F-87060 France; 2 UCA, INRAE, UMR PIAF, Clermont-Ferrand, F-63000 France; 3 UMR 0588 BioForA INRAE-ONF, Orléans, F-45075 France; 4 Université de Limoges, CAPTuR, UMR INSERM/CHU 1308, 2 Rue du Docteur Marcland, Limoges, F-87025 France

**Keywords:** Douglas-fir transcriptome, Heartwood formation, Seasonality, Wood tissues, Outer sapwood, Inner sapwood, Transition zone

## Abstract

**Objectives:**

Molecular cues linked to heartwood formation open new (complementary) perspectives to genetic breeding programs of Douglas-fir, a tree species largely cultivated in Europe for the natural durability and civil engineering properties of its wood.

**Data description:**

RNAs from a single genotype of Douglas-fir, extracted from three distinct wood zones (outer sapwood, inner sapwood and transition zone) at four vegetative seasons to generate an extensive RNA-seq dataset used to apprehend the in-wood dynamic and seasonality of heartwood formation in this hardwood model species.

Previously published data collected on somatic embryos of the same genotype could be merged with the present dataset to upgrade grade the Douglas-fir reference transcriptome.

## Objective

Douglas-fir (*Pseudotsuga menziesii*), a *Pinaceae* tree species originating from the North-Western parts of the United State and Canada, is currently considered in Europe as a major alternative to other endogenous conifers of economic importance [1] due to the natural durability and excellent building and civil engineering properties of its wood. Since its introduction in Europe, Douglas-firs have been selected for their growth qualities (trunk straightness, branching and rapid growth). To our knowledge, no comprehensive transcriptomic study focussed on the heartwood formation of Douglas-fir has been published until now [2–6]. For that purpose, 24-year-old trees from a comparative plantation plot located in Gimel (CFBL, France), managed by FCBA and planted at 1111 stems per hectare, were harvested for RNA-Seq analysis. Four provenances (1039, 80,145, 80,189 and 80,315) were selected for our study and one tree per provenance was felled each season (spring, summer, fall and winter), except for provenance 80,189 for which 3 trees were felled in winter and in summer. Genotypes were confirmed though the use of 11 nuclear microsatellite sequences previously characterised in Douglas-fir [7]. Indeed, these microsatellite markers confirmed that each genotype shared the same genetic heritage (data not shown). In the end, the 80,189 genotype was further selected on the basis of wood quality related phenotypic traits such as the colour and the relative surface area of its heartwood. RNA extractions were carried out from three distinct zones (growth rings) corresponding to outer sapwood (OSW), inner sapwood (ISW) and the transition zone (TZ) [8], the link to the file that describes the samples is given in Table 1 [9]. The major objective of our work is to characterise heartwood formation at the transcriptomic level.

Hence, we report here an update of the Douglas transcriptome project marked by the production of almost 400 million sequence reads focussed on wood and heartwood formation. This data complements the datasets describing the differentially expressed genes involved in somatic embryogenesis [10, 11]. Moreover, by combining both of these data (wood and somatic embryogenesis related cDNAs) with the data set already published on Douglas-fir seedlings [12–18], we propose to construct a new version of Douglas-fir reference transcriptome for the scientific community.

## Data description

A total of eight trees have been felled for this study, one in January and July, and three in April and October. In the field, wood slices were cut with a chainsaw and immediately frozen in liquid nitrogen. Total RNA was then extracted from freshly lyophilized disks. Three wood zones (growth rings) were harvested on each disk: the OSW that corresponds to the first ring under the cambial zone, the TZ, just located before heartwood, and the ISW representing the median ring between OSW and TZ. The rings of interest were isolated from the wood disc with a chisel, cut into fine bits and grinded with bench knife mill (4 × 5 s interspersed with a pause of 30 s) before being crushed into powder (granulometry of about 200 μm) by a ball milling machine (Labman Automation LTD, UK; Phenobois platform mill, INRAE Orléans) according to automated grinding sequences consisting of 6 grinding phases of 30 s interspersed with 50 s of pause.

The extraction of RNAs was first performed according to the protocol described by [16] slightly modified by [17]. The RNAs are then precipitated with 10 M LiCl (1:4, v/v). After 2 days at 4 °C, the RNAs are sedimented by centrifugation at 10,000 g for 30 min. The supernatant is removed. The RNA pellet is then dissolved in SSTE buffer, washes 2 times with chloroform/Isoamyl alcohol. After precipitation, RNA has been resuspended in 10 µl of DEPC-treated water.

The quantity and the purity of the total RNAs extracted were routinely determined with a micro-spectrophotometer (Nanodrop, ND-1000, ThermoScientific) and confirmed by electrophoresis using a Bioanalyzer 2100 (RNA 6000 Nano Kit, Agilent Technologies), a Bio-Rad Experion and a Fragment Analyzer (Advanced Analytical Technologies). Quality is given by the RQI (RNA Quality Index) for the Experion and by the RIN (RNA Integrity Number) for the other two.

The RNA-seq libraries were prepared using the Illumina strand-specific cDNA library kit according to the manufacturer’s guidelines. A total of 47 wood libraries were obtained before being sequenced using Illumina HiSeq 2500 sequencing technology with a maximum read length of 2 × 125 bp, the link to access the sequences deposited at the NCIB is given in Table 1 [19].

Quality trimming RNA reads obtained from sequencing where quality assessed using FastQC V1.11.4. Illumina adapter sequences left in reads were removed using CutAdapt v.1.2.1 (CutAdapt will search for a supplied list of adapters in all the reads, a minimum overlap of 15 bp between the adapter and the read is required). Low quality reads towards the 3’ and 5’ ends of the reads were trimmed with Trimmomatic v.0.3; reads were scanned with a 4 base wide sliding window and leading or trailing bases with average phred quality score lower than 20 were dropped. Reads with a length lower than 50 bp were also discarded, the links of the FastQC files of the different tissues are given in Table 1 [20–22].

Transcriptome de novo assembly. Digital normalization and transcriptome de novo assembly was conducted using the Trinity 2.0.6 software with a default k-mer size of 25. Trinity is composed of three different modules: Inchworm, Chrysalis and Butterfly. Inchworm builds a *k*-mer dictionary from the reads, which will lead to the construction of contigs. Chrysalis connects all overlapped contigs into components using a de Bruijn graph approach. In a final step, Butterfly simplifies all the generated graphs to report full-length transcripts and their alternatively spliced form. DeconSeq standalone version 0.4.3 was used to detect and remove sequence contaminations from the assembled transcriptome, using bacterial, fungal, plant, virus and other databases. DeconSeq was run with an alignment identity threshold of 95%.

**Table 1:** Overview of data files/data sets

Label	Name of data file/data set	File types (file extension)	Data repository and identifier (DOI or accession number)
Data file 1	Experimental_design	PDF file (.pdf)	*Recherche Data Gouv* repository : 10.57745/ELY4M6 [9]
Data set 1	Compressed RNA-seq files	Sequencing files (.fastq.bz2)	*NCBI Sequence Read Archive*: https://identifiers.org/ncbi/bioproject:PRJNA945886 [19]
Data file 2	MultiQC_trimmed_outer_sapwood	HTML file (.html)	*Recherche Data Gouv* repository: 10.57745/AGVBPI [20]
Data file 3	MultiQC_trimmed_inner_sapwood	HTML file (.html)	*Recherche Data Gouv* repository: https://doi.org/10.57745/DSMDCK [21]
Data file 4	MultiQC_trimmed_transition_zone	HTML file (.html)	*Recherche Data Gouv* repository: 10.57745/ERWUDJ [22]

## Limitations

- Due to the lethal sampling of Douglas-fir trees included in an on-going selection program, we only could have access to three biological repeats (trees) for two seasons (winter and summer) and one for the two other seasons.
- In some cases, especially for TZ, we have used two different rings of the same tree to have sufficient biomass for RNA extraction. In these cases, one ring was harvested at the base of the tree and the second at 1.30 m from the base of the trunk.

## Data Availability

The RNA-seq sequences are available in the National Center for Biotechnology Information (NCBI) with the BioProject accession number https://identifiers.org/ncbi/bioproject:PRJNA945886. The other data accessed on the Recherche Data Gouv repository under doi:10.57745/TZPTXE including [9, 20–22].

## Abbreviations

CFBL: Coopérative Forestière Bourgogne Limousin
FCBA: Forêt, Cellulose, Bois-construction et Ameublement
ISW: Inner Sapwood
OSW: Outer Sapwood
RNA-seq: RiboNucleic Acid - sequencing
k-mer: Substrings of length k
RQI: RNA Quality Index
RIN: RNA Integrity Number
TZ: Transition Zone
